# A medical student goes to the country of Nunavut

**DOI:** 10.36834/cmej.69040

**Published:** 2020-03-16

**Authors:** Thomsen D’Hont

**Affiliations:** 1 Faculty of Medicine, University of British Columbia, British Columbia, Canada

In my first year of medical school, I met the then Medical Director for Nunavut and was invited to do a fourth-year elective in Iqaluit. I did not forget about this offer, and as a fourth-year student I have successfully, with considerable effort and self-advocacy, signed up for (and completed) an “out-of-country” elective in Iqaluit, Nunavut this summer. This is the story of how challenging it is to sign up for a clinical elective in the Canadian territory of Nunavut, and how I believe the process should be changed to facilitate this important opportunity for clinical learning and physician recruitment in the territory.

I am a Métis person who grew up in the Northwest Territories (NWT), a territory that until 1999 included modern-day Nunavut. As an Indigenous medical student who is passionate about rural, remote, and northern medicine, and with many friends and a few familial ties to Nunavut, I jumped at the opportunity pursue some of my clinical training in the territory. However, when I started the process of applying for this opportunity, I quickly realized there was no available elective I could sign up for through the Association of Faculties of Medicine of Canada (AFMC) student portal.

For the uninitiated, the AFMC portal is the principal gateway for medical students across Canada to register for electives away from one’s home school. It was unveiled in 2014, and was designed to simplify the process of applying for visiting electives,^1^ allowing students to browse and apply for visiting electives on one centralized platform through the seventeen different medical schools across Canada. Students must sign up for pre-existing elective opportunities already listed in the portal and are dissuaded from setting up out-of-province electives through personal connections. Regions in Canada that don’t have a medical school, such as the NWT, typically have partnerships with medical schools that coordinate electives with them through memoranda of understanding (MOU). As an example, the University of Alberta has a MOU with the Government of the Northwest Territories to allow students to pursue electives in the NWT through the U of A. These electives are then listed on the portal. Unfortunately, in the case of Nunavut, there is no affiliated medical school and thus no elective listed. I spent hours on the phone and over email with Government of Nunavut officials and the medical programs that I had heard sent medical students to Nunavut. It was difficult to find answers. I learned that a few medical schools have MOUs for medical residentelectives and rotations in Nunavut, but not for medical students. Nunavut has their own elective application process, but because these electives are not associated with a medical school, students cannot get credit for them. As someone with few days of vacation, I would prefer not to put a rare recreational period towards an elective that is not for credit.

Through this process, I met with my 4^th^ year elective coordinator at UBC’s Northern Medical Program (NMP) in Prince George, BC, and was ultimately able to get the elective approved through an unconventional method. The work-around was to classify the elective as an “out-of-country” elective, which bypassed the AFMC portal. I had to fill out additional forms, and was required to write an essay about my “international” experience.

Although I am not from Nunavut, I am still a Northerner and am compelled to speak out on issues that impact education and recruitment of healthcare workers in Canada’s North. The literature shows that giving medical trainees experiences in rural and remote regions can improve recruitment and retention in these areas.^2^Anecdotally, this is indeed the case with Nunavut, where most of their recent returning locums and full-time staff are those who had rotations there as residents. Despite these successes, attracting physicians to work in Nunavut is still a challenge. This challenge is exacerbated when there are barriers preventing a population of potential future employees, namely medical students, from experiencing a place where they might consider working.

In terms of possible solutions, I encourage any medical school in Canada to collaborate with the Government of Nunavut to create an MOU allowing Canadian medical students to pursue elective training in the territory. Alternatively, the AFMC could recognize Nunavut’s current elective and allocate credit. Within the next few years I hope that an elective in Nunavut will be easily searchable on the AFMC portal, and that medical students can spend credited time in the Canadian territory of Nunavut, rather than in the “foreign country” of Nunavut. The training there is exceptional, with excellent one-on-one teaching and a young population with unique medical presentations, all in the setting of the tight-knit community of Iqaluit where visitors are welcomed with open arms.
